# Synergism of Fusobacterium periodonticum and N-nitrosamines promote the formation of EMT subtypes in ESCC by modulating Wnt3a palmitoylation

**DOI:** 10.1080/19490976.2024.2391521

**Published:** 2024-08-28

**Authors:** Mingjun Sun, Zhenyan Peng, Weitao Shen, Xinxin Guo, Yinghao Liao, Yang Huang, Ping Ye, Mohan Hu, Qiang Lin, Ran Liu

**Affiliations:** a Key Laboratory of Environmental Medicine Engineering, Ministry of Education, School of Public Health, Southeast University, Nanjing, China; b Department of Gastroenterology, Zhongda Hospital, Southeast University, Nanjing, China; c Department of Oncology, North China Petroleum Bureau General Hospital, Hebei Medical University, Renqiu, China

**Keywords:** Esophageal squamous cell carcinoma, N-Nitrosamines, Fusobacterium periodonticum, FadAL, Palmitoylation, Epithelial-mesenchymal transition

## Abstract

N-Nitrosamine disinfection by-products (NAs-DBPs) have been well proven for its role in esophageal carcinogenesis. However, the role of intratumoral microorganisms in esophageal squamous cell carcinoma (ESCC) has not yet been well explored in the context of exposure to NAs-DBPs. Here, the multi-omics integration reveals *F. periodonticum* (*Fp*) as “facilitators” is highly enriched in cancer tissues and promotes the epithelial mesenchymal transition (EMT)-like subtype formation of ESCC. We demonstrate that *Fp* potently drives de novo synthesis of fatty acids, migration, invasion and EMT phenotype through its unique FadAL adhesin. However, N-nitrosomethylbenzylamine upregulates the transcription level of FadAL. Mechanistically, co-immunoprecipitation coupled to mass spectrometry shows that FadAL interacts with FLOT1. Furthermore, FLOT1 activates PI3K-AKT/FASN signaling pathway, leading to triglyceride and palmitic acid (PA) accumulation. Innovatively, the results from the acyl-biotin exchange demonstrate that FadAL-mediated PA accumulation enhances Wnt3A palmitoylation on a conserved cysteine residue, Cys-77, and promotes Wnt3A membrane localization and the translocation of β-catenin into the nucleus, further activating Wnt3A/β-catenin axis and inducing EMT phenotype. We therefore propose a “microbiota-cancer cell subpopulation” interaction model in the highly heterogeneous tumor microenvironment. This study unveils a mechanism by which *Fp* can drive ESCC and identifies FadAL as a potential diagnostic and therapeutic target for ESCC.

## 1. Introduction

Esophageal cancer (EC), one of the most prevalent upper gastrointestinal cancers, significantly impacts the global cancer burden, particularly in Eastern Asia and Southern Africa. Annually, over 604,000 new EC cases are diagnosed worldwide, with esophageal squamous cell carcinoma (ESCC) accounting for approximately 90% of these cases, and the overall 5-year survival rate of ESCC patients remaining below 20%.^1^ Therefore, early screening and elimination of ESCC risk factors are necessary, but current research is still incomplete. Over recent decades, apart from non-modifiable risk factors such as age and family history in ESCC, studies have highlighted that the high incidence of ESCC is closely related to environmental risk factors, including biological factors, chemical carcinogens, living habits and drug factors.^2,3^ Specifically, polycyclic aromatic hydrocarbon exposure, hot food consumption, heavy smoking and excessive alcohol have all been implicated in the development of ESCC.^3^ Our previous study found that there was a certain correlation between N-nitrosamines in drinking water and ESCC in Huai’an area,^4^ and there was also a certain risk of microbial infections in this area.^5^ Notably, *Dubosiella* and *Peptostreptococcus* were closely associated with diethylnitrosamine-induced primary hepatocellular carcinoma;^6^ microcystins aggravated colorectal barrier disruption via changing gut microbiota, such as *Turicibacter* and *Clostridium*.^7^ These studies suggest that environmental factors tend to favor the development of certain specific flora, leading to disease initiation.

It is estimated that just 11 out of 10^12^ distinct microbial species on Earth have been identified as “human carcinogens” or “oncomicrobes”, contributing to 2.2 million new cases annually, roughly 13% of the total cancer cases.^8^ One of the best examples is in the context of a single microorganism: the well-established causal link between *Helicobacter pylori* and gastric cancer.^9,10^ Additionally, Parhi revealed *Fusobacterium nucleatum* (*Fn*) curbs tumor-infiltrating T cells and hastens the metastatic progression of breast cancer;^11^ And *Porphyromonas gingivalis* infection maintains oral cancer cell stemness.^12^ However, conventionally considered healthy esophagus is sterile or that the presence of any bacteria present is temporary, but it is now known to harbor a diverse microbiota.^3^ An investigation observed that genus *Haemophilus* and *Neisseria* present a significantly decreasing tendency, while *Streptococcus* has an increasing tendency with the progression of ESCC.^13^ Likewise, in Huai’an, a high-incidence area of ESCC attributed to N-Nitrosamine exposure, we preliminary found that the abundance of *F. periodonticum (Fp)* in tumor tissues was significantly higher than in para-carcinoma tissues.^3^ However, under N-Nitrosamine exposure conditions, whether *Fp* participated and how it played in carcinogenesis need to be investigated furtherly.

Increasing clues have indicated that molecularly distinct subtypes of ESCC differ in tumor microenvironment (TME). Liu et al. classified ESCC patients into four subtypes, including cell cycle pathway activation and immune suppression.^14^ Wang et al. identified three distinct TME signatures of ESCC (Wnt signaling pathway activation, inhibition of glycogen metabolism, and downregulation of neutrophil degranulation process).^15^ Noteworthy, a recent report highlights that Fn and *Porphyromonas gingivalis* induce cancer cell heterogeneity by altering distinct transcriptional programs that contribute to specific cell clusters.^16^ However, the process that pathogenic microbes induce the formation of different cellular subtypes may be activated by virulence genes.

As the subsequent functional studies found FadA genes from Fn increased the number of colorectal cancer cells that were in the S phase of the cell cycle through upregulating chk2;^17^ *Helicobacter pylori* release cagA, which induces carcinogenesis by disrupting cell polarity and promoting genetic instability.^18^ Additionally, *Porphyromonas gingivalis* also capable of upregulating inflammatory and oncogenic pathways in ESCC cells by activating TGF-β/Smad/YAP/TAZ signaling or GSK3β-mediated mtOXPHOS.^19,20^ However, there is a lack of research to elucidate the association between esophageal bacteria and molecular subtypes of ESCC tumors. Given this, we propose that the microbial virulence factors may also mediate the involvement of esophageal microbiota in ESCC development.

In the present study, we demonstrate that *Fp* is associated with the lipid metabolism disorder of EMT-like subtype of ESCC. *FadAL* as a virulence factor of *Fp* is upregulated by N-methyl-N-benzylnitrosamine (NMBzA) and binds to FLOT1 and activates PI3K/FASN signaling, leading to de novo synthesis of fatty acids as well as EMT phenotype. Further, we show that *Fp*-FadAL-mediated palmitic acid (PA) accumulation is both necessary and sufficient to enhance Wnt3A (Cys77) palmitoylation and activate β-catenin pathway, inducing the EMT phenotype. These findings suggest a causal role of *Fp* in advanced ESCC and highlight FadAL as a potential diagnostic and therapeutic target for ESCC.

## 2. Materials and methods

### 2.1. Bioinformatic database analysis

The gene expression and the decontaminated microbial composition data were retrieved from the Cancer Genome Atlas database (TCGA, https://portal.gdc.cancer.gov/) and the Cancer Microbiome Atlas database (TCMA, https://tcma.pratt.duke.edu/), respectively. A total of 47 cases were matched from TCGA and TCMA database for NMF cluster analysis to identify the ESCC subgroup, and TME signature of the ESCC subgroup (*n* = 47) was determined through GSVA.

### 2.2. Clinical data and specimens

This research received approval from the Institutional Review Board for Clinical Research at Zhongda hospital, affiliated with Southeast University (2021ZDKYSB004). Written informed consent was acquired from 102 ESCC patients who did not receive chemotherapy and/or radiotherapy and/or antibiotic treatment for at least 6 months. Surgically resected tumor tissues and para-carcinoma tissues (located 5 cm away from the tumor edge) from ESCC patients were collected under sterile conditions, immediately frozen in liquid nitrogen, and properly stored at −80°C for future use. Among these, 28 tumor and para-carcinoma tissues underwent qPCR analysis to determine *Fp* abundance, and while the remaining samples were used for qRT-PCR to assess human gene expression level. The characteristics of 28 and 74 ESCC patients were listed in Supplementary Table 1 and Table 2, respectively.

### 2.3. Bacterial strains and treatment

The *Fp* strain (ATCC-33693) was acquired from the American type culture collection. It was cultured in Brain-heart Infusion Broth supplemented with 5% defibrinated sheep blood (3.7%BHI, Hopebiol, China) and 0.1% L-Cysteine hydrochloride monohydrate (Sigma, USA) in an anaerobic jar (Millipore, Germany) at 37°C. The fourth generation of *Fp* strain (OD600 = 0.5) was cultured with 0 nmol/L,100 nmol/L, 1 μmol/L, 1 Mmol/L NMBzA for 48 h. The *E. coli* strain BL21 (Hopebiol, China) was cultivated on Luria-Bertani agar plate at 37°C. Planktonic growth of *Fp* was tracked by assessing the optical density (OD) at 600 nm.

### 2.4. Identification of virulence gene and preparation of recombinant proteins

The whole-genome sequences of *Fp* were acquired from GenBank database (https://ngdc.cncb.ac.cn/). A potential candidate virulence gene, named FadAL, was identified by comparing its amino acid sequences with those of *Fn* through NCBI-protein BLAST (https://www.ncbi.nlm.nih.gov/). The 400 bp FadAL gene was synthesized by Sangon Biotech Co., Ltd. (Shanghai, China) and subsequently cloned into the expression vector Pet28a. The plasmid (Pet28a-FadAL-His) was introduced into *E. coli BL21* and induced by 0.5 mmol/L isopropyl β-d-1-thiogalactopyranoside, leading to protein expression for 24 h at 37°C. Subsequently, recombinant FadAL was purified using Ni-NTA affinity chromatography and quantified using a BCA kit (Beyotime, China).

### 2.5. Cell culture and treatment

The human ESCC cell lines (EC109 and EC9706) and esophageal normal cells (Het-1A) were obtained from the American Type Culture Collection (ATCC, USA), and cultured in RPMI 1640 medium (Gibco, USA) medium containing 10% fetal bovine serum (FBS) and 1% penicillin and streptomycin (Gibco, USA) at 37°C in a 5% CO_2_ incubator. To create a bacteria-cell co-culture, ESCC cells (>85% confluency) were exposed to *Fp* at MOI of 1:1 for 12 h. For recombinant FadAL protein, ESCC cells (>70% confluency) were treated with FadAL at concentrations of 0, 0.1, 1, 10, 100 and 1000 ng/mL for 24 h, and cell viability was assessed at 450 nm using the Cholecystokinin octapeptide reagent (CCK-8, Beyotime, China).

### 2.6. Metabolomics and lipidomics analysis

The SCIEX Triple TOF 5600 system along with the Waters ACQUITY UPLC BEH C18 column was utilized for metabolomics and lipidomics analysis, adhering to our prior methodologies.^21,22^ Specifically, samples (1 × 10^7^ cells) were washed three times with pre-chilled PBS, then treated with 1 mL of pre-cooled −80°C methanol to halt cell activity before harvesting with scrapers. Subsequently, the cell suspension was centrifuged at 15,000 g for 15 min to obtain supernatant, which was further dried using a warm nitrogen flow. For LC-MS/MS analysis, the lipomics detection sample was dissolved in a mixture of 600 μL acetonitrile (ACN)/isopropanol (IPA)/deionized water (65:30:5, v/v/v) for lipidomics analysis, while metabolomics detection samples were dissolved in a mixture of 600ul Acetonitrile/Methanol (Merck, Darmstadt, Germany, 7:3, V/V). Subsequently, the raw data was processed and analyzed using progqen-esis QI software (Waters Corporation, Milford, USA). Data Processing Software, based on public data-bases, such as http://www.hmdb.ca/; https://ngdc.cncb.ac.cn/; http://www.lipidmaps.org/ and self-built data-bases.

### 2.7. Triglycerides and palmitic acid measurement

The triglycerides (TG) and PA concentrations in ESCC cells subjected to varied treatments were assessed following the manufacturer’s protocol of the human TG or PA ELISA kit (Jiangsu Meimian Industrial Co., Ltd). Specifically, over three million cells were collected and lysed by ultrasonication at 50 HZ (with 30 cycles of 5 s sonication separated by 20 s intervals) on ice. The supernatants were diluted 5 times and incubated with 100 μL HRP-conjugate reagent for 1 h at 37°C. After termination of the reaction, the absorbance was read at 450 nm using a multi-mode microplate reader (BioTek, USA). The BCA kit was employed to normalize the different samples to a uniform level.

### 2.8. Nile red stain

The Nile Red staining was performed to assess the level of intracellular lipid accumulation with transfected and/or FadAL treatment.^23^ ESCC cells were harvested, fixed with 4% paraformaldehyde for 10 min, and subsequently subjected to staining with 2 μg/L Nile Red (Yeasen Biotechnology (Shanghai) Co., Ltd.) for 30 min in the dark at 37°C. This was succeeded by a 10 min DAPI staining. Finally, the results were observed using a fluorescent microscope and flow cytometry.

### 2.9. Cell transfection

The siRNAs targeting FLOT1 (si-FLOT1), FASN (si-FASN) and the negative control (NC) were obtained from Sangon Biotech (Shanghai, China). Briefly, EC109 and EC9706 cells were initially seeded at a concentration of 2 × 10^5^ cells/well in 6-well plates and cultured for an additional 24 h. Then, the cells were transfected with 50 nmol/μL siRNA using the TransIntro® EL Transfection Reagent (Transgen, China) according to the manufacturer’s instruction. Interference efficiency was assessed via western blot analysis, and further experiments were performed 24 h after transfection. All sequences utilized in this study are available in Supplementary Table S3.

### 2.10. Transwell migration and invasion assay

As previously described,^4^ the cell migration and invasion abilities were evaluated using 8.0 μm Transwell Chamber (Corning, USA). Briefly, cells that were transfected and/or treated with FadAL (5.0 × 10^5^ cells/well for invasion assay, 2.5 × 10^5^ cells/well for migration assay) were suspended in 200 μL of serum-free RPMI-1640 and placed in the upper chambers, while the lower chamber was filled with RPMI-1640 containing 20% FBS. After 24 h of incubation, cells were fixed with 95% ethanol for 10 min and stained with 0.1% crystal violet dye. The number of migrated cells was counted using FSX100 Bioimaging navigator (Olympus Corporation, Tokyo). The cell invasion assay, which required Matrigel coating, was conducted in a similar manner.

### 2.11. Co-IP analysis

The ESCC cell protein was extracted using RIPA Lysis Buffer containing protease inhibitor and phosphatase inhibitor (Epizyme, China). The extracted proteins’ concentrations were measured by BCA kit after centrifuging cell suspensions to eliminate insoluble components. The equal lysates (1.5 mg, containing 3 μg recombinant FadAL protein) were then incubated with IP-graded primary antibody (anti-FLOT1, anti-His-Tag or IgG) on a shaking platform at 4°C overnight. Following this, the protein A/G agarose beads (MedChemExpress, USA) were added and maintained for 6 h. Lastly, the protein samples were denatured and analyzed through western blot. Antibody information is detailed in Supplementary Table S4.

### 2.12. Palmitoylation assays

Protein palmitoylation levels were determined using the Acyl-biotinyl exchange (ABE) assay.^24^ In brief, cells were lysed with RIPA Lysis Buffer (Epizyme, China) supplemented with 1% (v/v) protease inhibitor (PI) and 10 mM N-Ethylmaleimide (Solarbio, China). This lysate was then centrifuged (12,000 g/15 min, 4°C) to gather total supernatant. Afterwards, 1.5 mg of total protein was diluted in lysis buffer (LB, 1% (v/v) Triton X‐100, 1% (v/v) PI,1 mM EDTA, 50 mM Tris‐HCL, 150 mM NaCl, pH7.5) for chloroform-methanol (CM) precipitation. Subsequently, the dried proteins were re-suspended in 200 μL SDS buffer (SB, 20% SDS, 1 M Tris-HCl, 0.5 M EDTA, pH 7.5) and 800 μL LB containing 1% (v/v) PI, then incubated at 4°C overnight on a shaker. After three CM precipitations, each sample was separated into two sections (1/2 for -HAM, 1/2 for +HAM) and incubated at 4°C for 3 h. The sample was then treated with transformation buffer (TB) and LB and incubated with 25 μL BeyoMag™ Streptavidin Magnetic Beads (Beyotime, China) for 6 h at 4°C. Finally, resin-captured proteins were eluted with LB buffer (1% (v/v) β-mercaptoethanol, 20% (v/v) Loading buffer) and were performed to Western blot.

### 2.13. Xenograft mouse model

The animal protocol was approved by the Institutional Animal Care and Use Committee of Southeast University (#20240304001). An inoculum of 8.0 × 10^6^ Ec109 cells was injected subcutaneously and bilaterally into 4-week-old female NCG mice (6 per group) as our previously described.^25^ When the tumor volume reached approximately 150 mm^3^, mice were randomized to receive either *Fp* at 8.0 × 10^6^ colony-forming units (CFU) or PBS at each inoculation site, once a week, three times in total. Subsequently, the fluorescence in situ hybridization (FISH), hematoxylin and eosin (H&E) staining and immunohistochemistry (IHC) were performed.

### 2.14. FISH assay

FISH was performed as described previously.^25^ *Fp* Alexa Fluor 488-conjugated specific probe (TTCTATCAGGTCATAGTCTAAACTTTTTTATCA) was labeled with Spectrum-Green (GENERAY Biotechnology). Briefly, xenograft tumor tissues were fixed in Carnoy’s solution overnight and embedded in paraffin; 5 μm-thick sections were hybridized in the hybridization buffer. Pre-warmed hybridization buffer with *Fp*-specific probe (0.25 pmol/mL) and used to the tissue sections. After incubation for 5 h in a dark humid chamber at 46°C, each of the slides was rinsed with sterile double-distilled water, air-dried in the dark and mounted with 1 ng/mL DAPI (Yeasen Biotechnology (Shanghai) Co., Ltd.). Images were acquired on an inverted fluorescent microscope.

### 2.15. IHC assay

The nude mice xenograft tissues were fixed with 4% paraformaldehyde for 24 h and then embedded in paraffin for making slices with a thickness of 5 µm. After deparaffinization and rehydration, the tissues were then blocked with endogenous peroxidase for 15 min, then incubate tissues with TBST containing 0.5% primary antibody (HUABIO, China) (E-cadherin (ET1607–75), N-cadherin (ET1607–37), FLOT1 (ET7107-82), Wnt3A (HA500193) and FASN (ET1701-91)) at 4°C overnight. Subsequently, samples were incubated with an HRP-IgG secondary antibody at RT for 1 h and then incubated with streptavidin peroxidase complex. Finally, samples were stained by diaminobenzidine and counterstained with hematoxylin and analyzed with a light microscope.

### 2.16. DNA/RNA isolation and quantitative real-time PCR (RT-qPCR)

Genomic DNAs were extracted from esophageal tissue samples using TIANamp Bacteria DNA Kit (TIANGEN, China). Total RNA from ESCC cell lines (EC109 and EC9706) or esophageal tissue samples was extracted using Trizol reagent. The RNA was then reverse transcribed into cDNA via HiScript II 1st Strand cDNA Synthesis Kit (Vazyme, China) following the manufacturer’s protocol and stored at −20°C. Subsequently, the RT-qPCR was performed using ChamQ SYBR qPCR Master Mix (Vazyme, China) in a StepOne Plus System (Applied Biosystems, USA) following standard procedures. The β-actin and universal *Eubacteria* 16S served as an internal control for human genes and *Fp* quantification, respectively. All primer sequences were shown in Supplementary Table S4.

### 2.17. Western blot

Total cellular proteins were extracted using RIPA Lysis Buffer, and the concentrations were measured via BCA method. Then, 20–40 μg protein was separated by 10% or 12% SDS-PAGE and transferred onto 0.45 μm PVDF membrane (Millipore, USA). The membranes were blocked with TBST containing 5% nonfat powdered milk (Sangon Biotech, China) for 2 h at RT and incubated with primary antibodies at 4°C overnight. After five washes with TBST, the bands were incubated with secondary antibody at RT for 2 h and detected using Enhanced Chemiluminescence Kit (Epizyme, China) in Tanon-5200 Chemiluminescence gel imaging system (Tanon, China). The information of antibodies was listed in Supplementary Table S5.

### 2.18. Statistical analysis

All statistical data were presented as the mean ± Standard Deviation (x ± SD) and were representative of a minimum of three independent experiments. Statistical analyses involving differences between variables were conducted using paired Student’s t-test, independent sample t-test, and χ2-test via SPSS 16.0 software or Prism 7.04 software. A p-value of <0.05 was considered statistically significant.

## 3. Results

### 3.1. Fp is enriched in EMT-subtype of ESCC and associated with lipid metabolism

**Figure 1.**
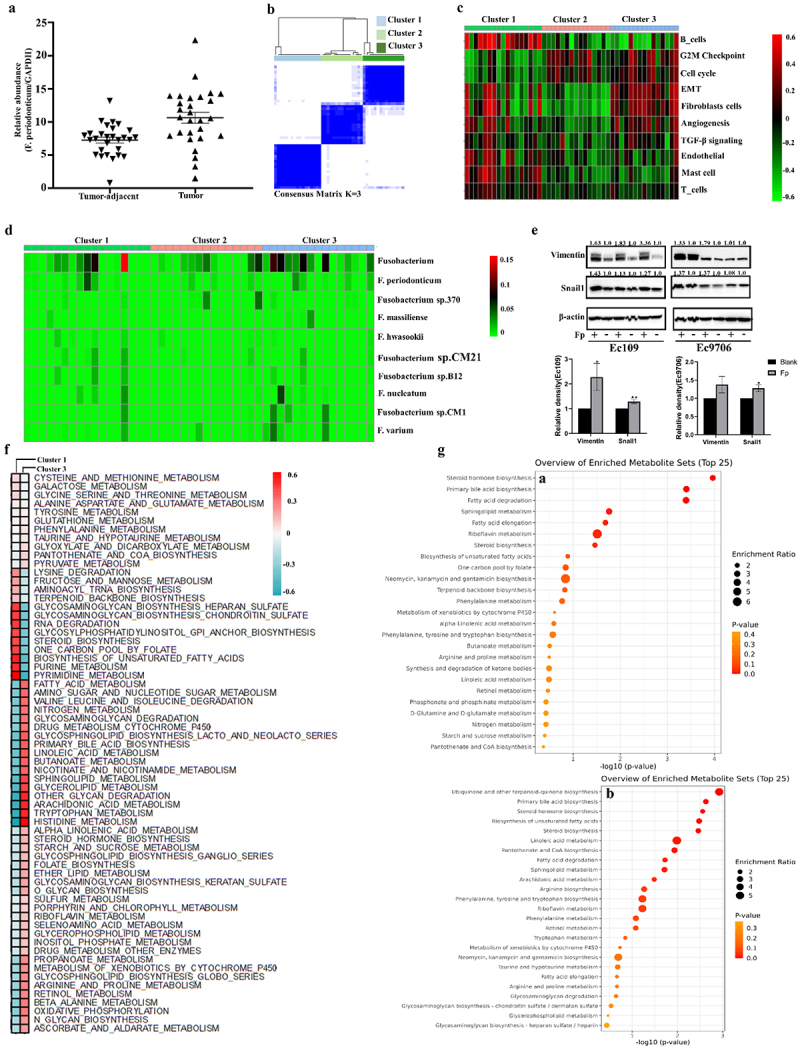
*Fp is enriched in emt-subtype of ESCC and associated with lipid metabolism*. (a) The relative abundance of Fp in the ESCC tissues was determined by qRT-pcr (*n* = 28). (b) Based on TME signatures, ESCC patients (*n* = 47) were divided into three clusters by the NMF algorithm (K = 3). (c) Heatmap describing the three clustering results based on TME signatures. (d) Heatmap showing the differentially abundant of Fusobacterium in clusters 1, 2 and 3. (e) Western blots were performed, and relative protein levels of Snail1 and vimentin in the ESCC cells with F p infection were determined (*n* = 3). (f) The metabolic characteristic of clusters 1 and 3 were assess by GSEA. (g) Enrichment analysis of differentially metabolites with metaboanalyst 5.0, ranking by enrichment ratio (a: Ec109; b: Ec9706). *p < 0.05 compared with controls.

Based on our previous 16s rDNA sequencing data of ESCC tissues,^3^ further, the RT-qPCR confirmed a notably higher abundance of *Fp* at unit cell level in cancer tissue compared to adjacent tissues (Figure 1(a)). Next, to decipher the relationship between *Fp* and ESCC tumor molecular subtypes, we included 47 RNA-seq datasets of ESCC from the intersection of TCGA and TCMA. Utilizing the GSVA analysis for the TME signatures in ESCC (Table S4), patients were segregated into three clusters employing the NMF algorithm (Figure 1(a,b)). Simultaneously, Cluster 1, Cluster 2 and Cluster 3 were defined as immunity-like state, cell cycle like state and EMT-like state, respectively (Figure 1(c)). We also observed that *Fusobacterium* was enriched in EMT state characterized by a high metastatic burden (Figure 1(d); Fig. S1B-SD), and the detection rate of *Fp* was highest in the EMT state (Figure 1(e)). In vitro, the preliminary findings indicated that *Fp* infection led to an upregulation in the expression of Snail1 and Vimentin (Figure 1(e)). Thus, these findings indicate an association between the increased abundance of *Fp* and the EMT phenotype of ESCC.

Multiple pieces of evidence support the pivotal role of metabolic reprogramming in the initiation and maintenance of EMT.^26^ Here, we further deciphered the metabolic characteristics of the molecular subtypes of ESCC tumors, and observed significant enrichment in sphingolipid, fatty acid, and glycerolipid metabolism in the EMT-subtype of ESCC (Figure 1(f)). Additionally, we systematically analyzed the cellular metabolic profile utilizing LC-MS/MS during *Fp* infection. The scoring plots of OPLS-DA models for both negative and positive modes revealed pronounced separation between blank group and *Fp-*infected group (Fig. S1F-SI). Particularly noteworthy, the top 5 enriched metabolic pathways primarily revolve around lipid metabolism, including steroid hormone biosynthesis, primary bile acid biosynthesis, unsaturated fatty acid biosynthesis, sphingolipid metabolism and fatty acid elongation (Figure 1(g); Fig. S1J-M). Collectively, these findings indicate that the lipid metabolism disruption facilitated by *Fp* may induce the creation of EMT subtypes.

### 3.2. N-Nitrosamines upregulate fp adhesin FadAL leading to EMT phenotype

**Figure 2.**
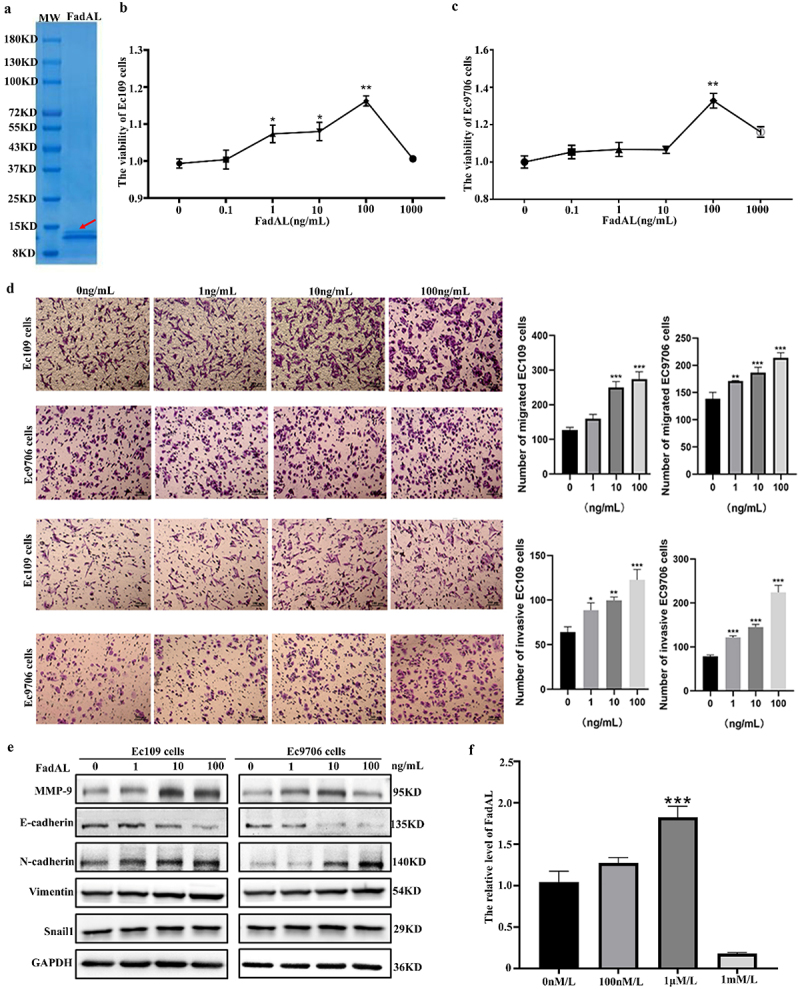
*Fp-*FadAL induces EMT phenotype of ESCC cells. (a) Analysis of the FadAL protein preparation and purification on 12% SDS – PAGE gels by Coomassie blue R250 staining. (b–c) The cell viability was determined by the CCK-8 assay (*n* = 6). (d) Transwell assays were performed to assess and quantify the migration and invasion of ESCC cells with FadAL treatment (*n* = 3). (e) Western blots were performed, and relative protein levels of Snail1, N-cadherin, Vimentin, and E-cadherin were determined (*n* = 6). (f) FadAL mRNA levels in *Fp* with NMBzA treatment (*n* = 3). *p < 0.05, *p < 0.01 and ***p < 0.001.

Next, to investigate the potential carcinogenic effects of *Fp*, by comparing the amino acid sequences with *Fp* based on NCBI-protein BLAST, we identified FadAL as a virulence factor that may have carcinogenic properties. FadAL consists of 132 amino-acid residues, belonging to the FadA superfamily (Fig. S2A). However, the homology index between FadAL and the FadA of *Fn* was only 37.4% (Fig. S2B), implying that FadAL may have distinct functions from FadA. Subsequently, to explore the functions and mechanism of FadAL, the recombinant FadAL-His tag fusion protein was prepared and purified (Fig. S2C). Coomassie brilliant blue staining on 12% SDS-PAGE confirmed molecular weight of the FadAL protein was 13 kDa (Figure 2(a)). We further investigated the impact of FadAL on ESCC cells. The CCK-8 assay was performed following treatment of ESCC cells with 0–1 μg/mL of FadAL recombinant protein for 24 h. Compared with the control, the cell viability was increased with 0.1–100 ng/mL FadAL recombinant protein, peaking at 100 ng/mL (Figure 2(b–c)). Therefore, focusing on the toxic effects of low-concentration FadAL, we selected 0, 1, 10, and 100 ng/mL for further experiments. After exposing Ec109 and Ec9706 cells to these concentrations for 24 h, the Transwell assay revealed that FadAL, compared to the control, concentration-dependently enhanced the migration and invasion ability of cancer cells (Figure 2(d)). At the molecular level, we analyzed the expression levels of EMT-related proteins and observed that FadAL upregulated MMP9, N-cadherin, Vimentin, and Snail1 expression while downregulating E-cadherin levels (Figure 2(e)). Considering our previous investigation results that the N-Nitrosamines were higher in ESCC patients compared with the healthy people,^4^ we also investigated the relation between the *Fp* and N-nitrosamines, and found 100 μM/L NMBzA significantly upregulated the transcription level of FadAL (Figure 2(f)). These findings indicate that N-nitrosamines mediated FadAL expression can promote the malignant progression of ESCC.

### 3.3. Fp-FadAL promotes the de novo synthesis of fatty acids in ESCC cells

**Figure 3.**
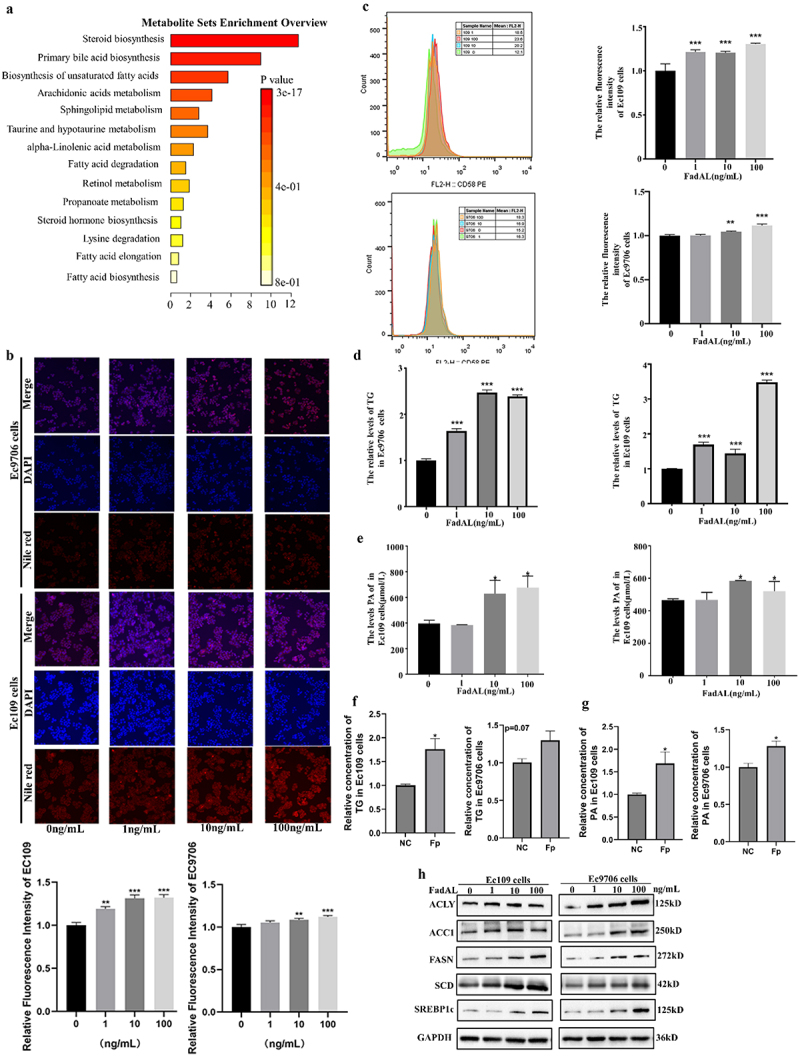
*Fp*-FadAL promotes lipid accumulation of ESCC cells. (a) Functional annotation of differentially metabolites with metaboanalyst 5.0, ranking by enrichment ratio. (b–c) Nile Red staining were used to verify the lipid accumulation of ESCC cells by the fluorescence microscope (B, *n* = 6) and flow cytometry (C, *n* = 6). (d-e) The TG (D) and PA(E) levels in ESCC cells treated with FadAL were assessed by ELISA (*n* = 6). (f–g) The TG (F) and PA(G) levels in ESCC cells infected with *Fp* were assessed by ELISA (*n* = 6). (h) Western blots were performed, and relative protein levels of ACLY, ACC1, FASN, SCD and SREBP1 were determined (*n* = 3). *p < 0.05, *p < 0.01 and ***p < 0.001.

Considering the relationship between *Fp* and lipid metabolism, the lipidomic profile of cells was further systematically analyzed by LC-MS/MS to describe the dysfunction of lipid metabolism during the formation of EMT induced by exposure to 0 or 100 ng/mL FadAL recombinant protein for 24 h. The scoring plots of OPLS-DA models for both negative and positive modes revealed pronounced separation between control group groups and the 100 ng/mL FadAL-treated group, suggesting the clustering of different treatment groups and demonstrating good model fit and predictive performance (Fig. S3A). Importantly, a total of 60 upregulated and 70 downregulated lipid metabolites were identified in 100 ng/mL FadAL-treated group (*p* < 0.05; FC > 1.5) (Fig. S3B&C). Next, an enrichment analysis was performed on the screened differential metabolites, highlighting the top 3 enriched metabolic pathways: steroid biosynthesis, primary bile acid biosynthesis, and unsaturated fatty acid biosynthesis (Figure 3(a); Fig. S3D). Therefore, we focused on the unsaturated fatty acid biosynthesis pathways due to the overlapping metabolic pathways between the data of MS and GSVA. Further, considering different assays with varying advantages and drawbacks regarding sensitivity, limit of detection, and breadth of coverage, we performed the Nile red staining to confirm the effect of *Fp* and FadAL on the lipid metabolism of ESCC. The Nile red staining results revealed that FadAL recombinant protein promoted lipid accumulation Ec109 and Ec9706 cells in a dose–response manner (Figure 3(b–c)), which is consistent with *Fp* infection group (Fig. S3E). Additionally, ELISA results showed that TG levels were higher in *Fp* infection or FadAL treatment group compared to the control group (Figure 3(d–f)). Remarkably, studies have confirmed that fatty acid biosynthesis begins with acetyl-CoA and results in PA formation.^27^ Subsequent PA ELISA experiments confirmed that *Fp*-FadAL also significantly induced PA accumulation (Figure 3(e–g)). At the molecular level, western blot analysis demonstrated that FadAL exposure upregulated the expression levels of key genes in the fatty acid synthesis pathway, including ACLY, ACC1, FASN, SCD and transcription factor SREBP1 (Figure 3(h)). These results show that, for ESCC cells, FadAL induces lipid accumulation and higher PA levels may be key mechanisms by which *Fp* promotes the EMT phenotype.

### 3.4. FadAL binds to FLOT1 to induce fatty acid accumulation by PI3K-AKT/FASN axis

**Figure 4.**
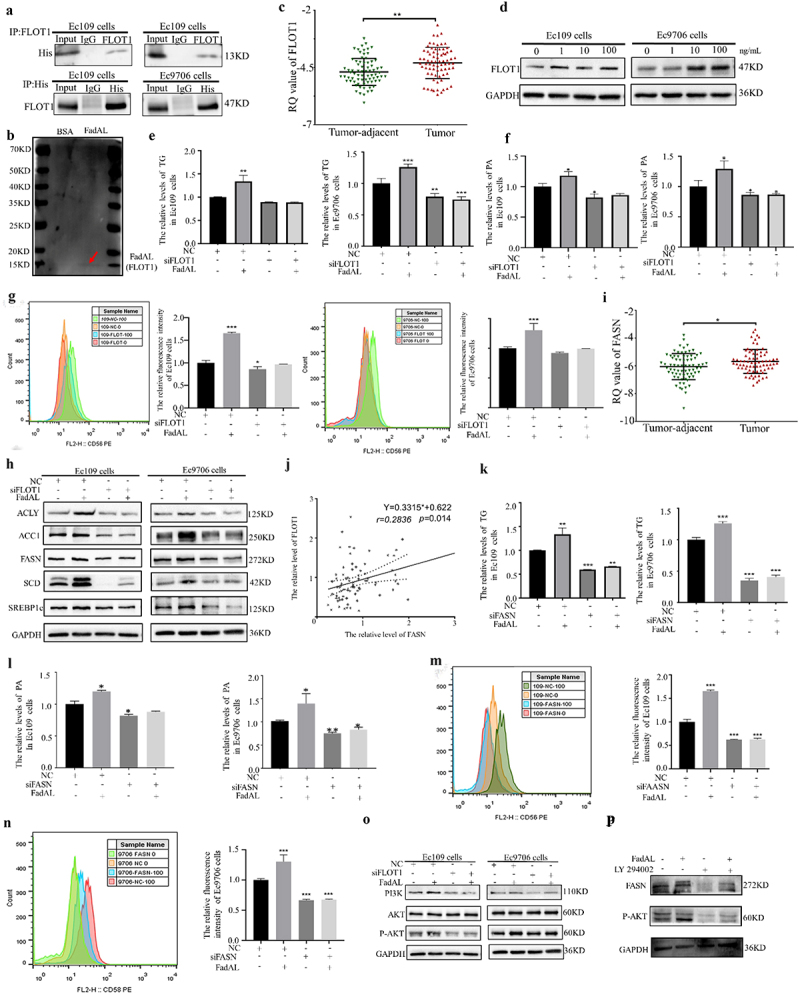
FadAL binds to FLOT1 to induces fatty acid accumulation by PI3K-AKT/FASN axis. (a–b) The protein interaction between FadAL-His and FLOT1 was verified by CO-IP(A) and Far-Western blot(b), respectively. (c) FLOT1 levels of parental adjacent and tumor ESCC tissue were assessed by qRT-PCR (*n* = 74). (d) Western blots were performed, and FLOT1 protein level in ESCC cells treated with different concentrations of FadAL (0, 1, 10, or 100 ng/mL) were determined (*n* = 3). (e–f) The levels of TG (E) and PA(F) in ESCC cells treated with 100 ng/mL FadAL and/or siFLOT1 were assessed by ELISA (*n* = 6). (g) Nile Red staining was employed to verify the lipid accumulation of ESCC cells with 100 ng/mL FadAL and/or siFlot1using the flow cytometry (*n* = 6). (h) The relative protein levels of ACLY, ACC1, FASN, SCD and SREBP1 were determined by Western blots in ESCC cells treated with 100 ng/mL FadAL and/or siFLOT1 (*n* = 6). (i) FASN mRNA levels of parental adjacent and tumor ESCC tissue were assessed by qRT-PCR (*n* = 74). (j) The correlation between the expression level of FLOT1 and FASN in ESCC tissues (*n* = 74). (k–l) The levels of TG (K) and PA(L) in ESCC cells treated with 100 ng/mL FadAL and/or siFASN were assessed by ELISA (*n* = 6). (m-n) Nile Red staining was employed to verify the lipid accumulation of ESCC cells with 100 ng/mL FadAL and/or si-FASN using the flow cytometry (*n* = 6). (o) The protein levels of PI3K, AKT and p-akt were determined by Western blots in ESCC cells treated with 100 ng/mL FadAL and/or siFLOT1 (*n* = 3). (p) The protein levels of PI3K, AKT and FASN were determined by Western blots in ESCC cells treated with 100 ng/mL FadAL and/or LY294002 (*n* = 3). *p < 0.05, *p < 0.01 and ***p < 0.001.

ESCC cell receptors for FadAL were identified to investigate how FadAL enhances the fatty acid accumulation in ESCC cells. It was previously reported that *Fn*-FadA binds to E-cadherin on CRC cells,^28^ leading to the hypothesis that FadAL might also bind to E-cadherin. However, subsequent molecular docking simulations showed the no binding site between FadAL and E-cadherin. Further, Co-IP/MS identified FLOT1 specifically interacting with FadAL on the surface of ESCC cells (Unpublished). Subsequently, FadAL binding to FLOT1 was indirectly and directly tested by molecular docking simulations (Fig. S4A-C), Co-IP (Figure 4(a)) and Far-Western Blot (Figure 4(b)), respectively. Moreover, FLOT1 mRNA expression was significantly higher in tumor tissues compared to adjacent non-tumor tissues (Figure 4(c)). Clinically, patients with high expression of FLOT1 were correlated with low-grade ESCC differentiation (Table S2). In vitro, treating Ec109 and Ec9706 cells with 0, 1, 10, or 100 ng/mL of FadAL recombinant protein for 24 h demonstrated a concentration-dependent upregulation of FLOT1 protein expression (Figure 4(d)). These results suggested that FadAL binds to FLOT1, activating its expression in ESCC.

Next, to further explore the role of FadAL binding to FLOT1 in these processes, the low-expressed FLOT1 cancer cell lines were successfully constructed (Fig. S4D). Subsequently, ELISA data confirmed that FLOT1 knockdown eliminated the cumulative effects of FadAL recombinant protein on TG and PA in ESCC cells (Figure 4(e–f)). Similarly, flow cytometry experiments showed that FadAL-induced lipid accumulation was curbed after transfecting si-FLOT1 in Ec109 and Ec9706 cells (Figure 4(g)). In addition, western blot analysis showed that, compared with the FadAL treatment group, the group with knockdown of FLOT1 had decreased ACLY, ACC1, FASN, SCD and SREBP1 expression (Figure 4(h)). These data confirm that FLOT1 is involved in FadAL-mediated fatty acid accumulation of ESCC cells.

FASN, the sole human lipogenic enzyme for de novo fatty acid synthesis, is responsible for endogenous fatty acid synthesis.^29^ Clinical data revealed significantly higher FASN expression in ESCC tissues compared to normal tissues (Figure 4(i); Fig. S4E), and positively correlating with FLOT1 expression (Figure 4(j)). By constructing a low-expressed FASN cancer cell line (Fig.S4F), it was found that suppressing of FASN almost completely attenuated intracellular TG and PA accumulation (Figure 4(k–n)). To further explore the signaling pathways of FadAL-FLOT1 regulating FASN, we analyzed the protein levels of PI3K, p-Akt, and Akt. While there was no significant difference in total AKT, PI3K and p-Akt levels increased after 100 ng/mL FadAL treatment (Figure 4(o)). Western blot analysis showed that, compared with the FadAL treatment group, the group with knockdown of FLOT1 had reduced PI3K and p-Akt expression (Figure 4(o)). Importantly, LY294002, a PI3K inhibitor, suppressed the FadAL-medicated FASN upregulation (Figure 4(p)). Taken together, these results indicate that in ESCC cells, FadAL, targeting FLOT1, contributes to fatty acid accumulation via the PI3K-Akt/FASN pathway.

### 3.5. PA-mediated Wnt3A palmitoylation activate Wnt3A/β-catenin pathway to induces EMT phenotype

**Figure 5.**
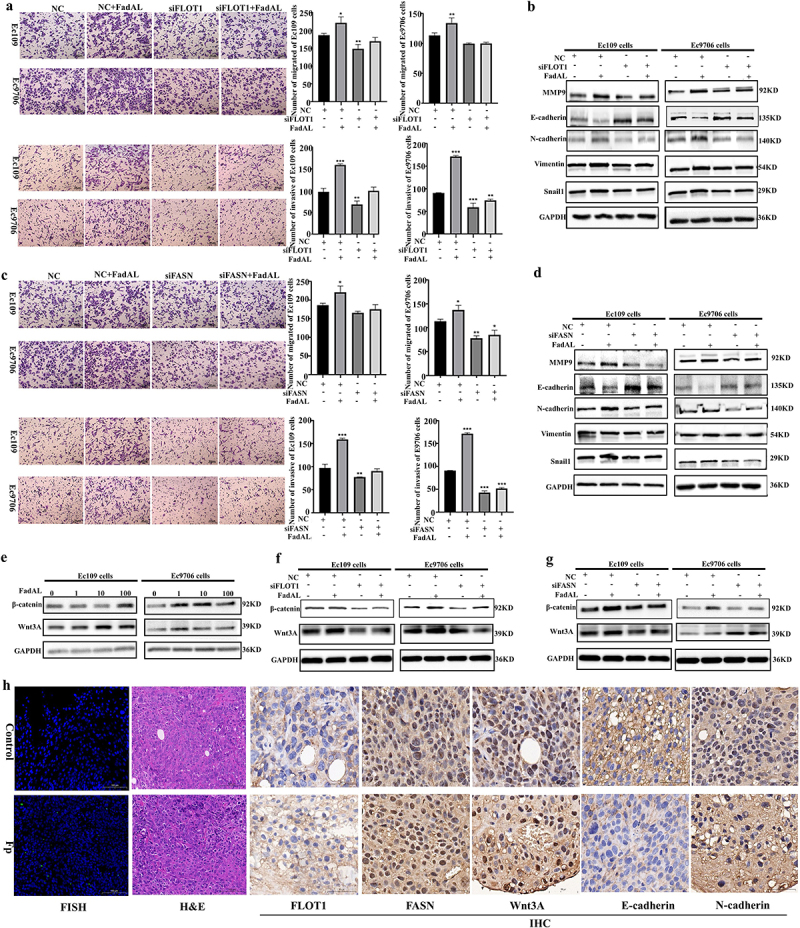
*Fp*-FadAL induces EMT phenotype by FLOT1/FASN/Wnt3A axis in vitro and in vivo. (a) Transwell assays were performed to assess and quantify the migration and invasion of ESCC cells with 100 ng/mL FadAL and/or siFLOT1 treatment (*n* = 3). (b) Western blots were performed, and relative protein levels of MMP9, Snail1, N-cadherin, Vimentin, and E-cadherin were determined in ESCC cells with 100 ng/mL FadAL and/or siFLOT1 treatment (*n* = 3). (c) Transwell assays were performed to assess and quantify the migration and invasion of ESCC cells with 100 ng/mL FadAL and/or siFLOT1 treatment (*n* = 3). (d) Western blots were performed, and relative protein levels of MMP9, Snail1, N-cadherin, Vimentin, and E-cadherin were determined in ESCC cells with 100 ng/mL FadAL and/or siFASN treatment (*n* = 3). (e) The protein levels of Wnt3A and β-catenin were determined by Western blots in ESCC cells treated with different concentrations of FadAL (0, 1, 10, or 100 ng/mL) (*n* = 3). (f–g) The protein levels of Wnt3A and β-catenin were determined by Western blots in ESCC cells treated with 100 ng/mL FadAL and/or siFLOT1(F) and/or siFASN(G) treatment (*n* = 3). (h) FISH staining, H&E staining and immunohistochemical staining of tumors from mice administered *Fp* treatments. *p < 0.05, *p < 0.01 and ***p < 0.001.

We explore the role of *Fp*-FadAL/FLOT1/FASN axis mediated fatty acid accumulation in promoting the EMT process of ESCC cells. As expected, si-FLOT1 or si-FASN effectively inhibited the 100 ng/mL FadAL-induced migration and invasion of Ec109 and Ec9706 cells (Figure 5(a,b)). Consistent with these observations, si-FLOT1 also blocked tumor cell migration and invasion stimulated by *Fp* infection (Fig. S5A). In addition, FadAL recombinant protein triggered-elevated MMP-9, N-cadherin, Vimentin and Snail, and decreased E-cadherin were rescued by si-FLOT1 or si-FASN (Figure 5(c,d)). Next, we further confirmed the potential mechanisms of FASN alteration in FadAL/FLOT1-triggered EMT phenotype. In a variety of solid tumors, Wnt/β-catenin axis is constitutively active and promotes EMT.^30^ To test this possibility, we performed FadAL treatment or/and si-FLOT1 or/and si-FASN transfection. The results demonstrated a significant elevation in Wnt3A and β-catenin protein levels in ESCC cells treated with FadAL compared to the control group (Figure 5(e)). Interestingly, a decrease in Wnt3A and β-catenin expression was observed with FLOT1 or FASN knockdown in Ec109 and Ec9706 cells (Figure 5(f–g)). To verify the roles of *Fp* in ESCC cells in vivo, Ec109 cells were subcutaneously inoculated into NCG mice. As shown in Figure 5(h), *Fp* was enriched in xenograft tumor tissues. We also observed enhanced staining of FLOT1, FASN, N-cadherin and Wnt3A in *Fp*-treated xenograft tissues, while E-cadherin expression was decreased (Figure 5(h); Fig. S5B). These results suggest that *Fp-*FadAL plays a key role in the EMT phenotype of ESCC cell in vitro and in vivo.

**Figure 6.**
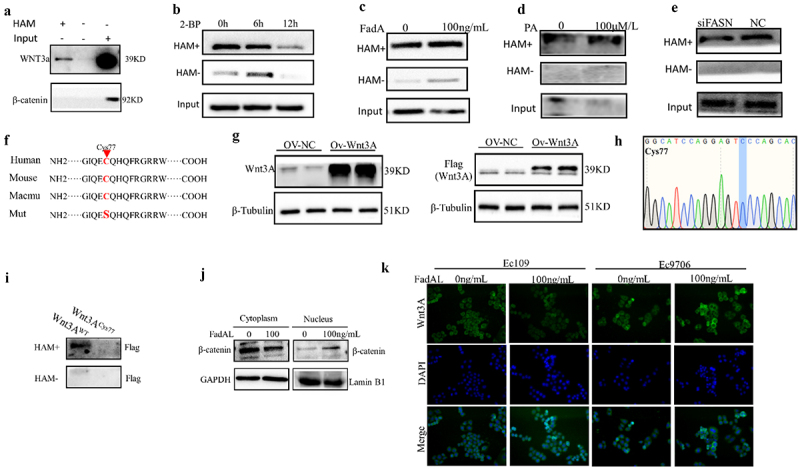
Pa-mediated Wnt3A palmitoylation activate Wnt3A/β-catenin pathway to induces EMT phenotype. (a–e) ABE assay was performed, and Wnt3A palmitoylation level were determined in Het-1A cell (a), and Ec109 cells treated with 2-BP (b), or 100 ng/mL (c) or siFASN (d) or PA (e) treatment. (f) Predicted position of palmitoylation site on Wnt3A in Homo sapiens, mouse and macmu using Swiss-Palm software. (g) Western blots were performed, and relative protein levels of Wnt3A or flag were determined in pcDNA3.1-flag-Wnt3A plasmid group (*n* = 3). (h) sequence of back-splicing site of pcDNA3.1-flag-Wnt3Acys77 plasmid verified by Sanger sequencing. (i) ABE assay was performed, and Wnt3A palmitoylation level were determined in Ec109 cells treated with Wnt3AWT or Wnt3ACys77 treatment. (j) The β-catenin expression was determined by western blot in the nucleus and in the cytoplasm of ESCC cells with 100 ng/mL FadAL treatment. (k) After 100ng/mL FadAL treatment, the subcellular localization of Wnt3A was confirmed by immunofluorescence in the ESCC cells.

Notably, latest research suggested that PA accumulation enhanced β-catenin palmitoylation and stabilization to promote CRC progression.^24^ To analyze the effects of PA on β-catenin palmitoylation in ESCC, ABE and western blot assays were performed. Results indicated that β-catenin did not exhibit palmitoylation features in Het-1A cells, while Wnt3A did (Figure 6(a)). The 2-bromopalmitate (2-BP), a palmitoylation inhibitor, significantly inhibited the Wnt3A palmitoylation of ESCC cells in a time-dependent manner (0 h, 6 h,12 h) (Figure 6(b)). Additionally, we further identified the palmitoylation level of Wnt3A after 100 ng/mL FadAL recombinant protein or 100 μM/mL PA or si-FASN treatment. In this analysis, the Wnt3A palmitoylation level was significantly upregulated with FadAL or PA treatment (Figure 6c–d), while decreased in cells with FASN downregulation (Figure 6(e)), supporting the hypothesis that FadAL/FASN-mediated PA accumulation can enhance Wnt3A palmitoylation modification. Moreover, we analyzed the S-palmitoylation sites of Wnt3A using Swisspalm and previous research.^31^ As shown in Figure 6(f), Cys77 is well conserved across species. The wild-type pcDNA3.1-Flag-Wnt3A plasmid (Wnt3A^Flag-WT^) served as a template for site-directed mutagenesis (Figure 6(g-h)). The point mutation plasmids Wnt3A^Flag-Cys77^was constructed and expressed in Ec109 cells. Remarkably, Wnt3A^Flag-Cys77^ showed a significantly reduced level of palmitoylation in FadAL treatment group compared to Wnt3A^Flag-WT^ (Figure 6(i)). Taken together, our data show that Wnt3A is palmitoylated that cysteines 77 is the main sites of palmitoylation. On the other hand, the results of immunofluorescence and western blot showed that FadAL treatment significantly increased the β-catenin nuclear translocation (Figure 6(j)) and Wnt3A membrane localization (Figure 6K). Taken together, the results suggested that FASN/PA-mediated Wnt3A (Cys77) palmitoylation activates Wnt3A/β-catenin pathway, leading to the induction of EMT phenotype.

*p < 0.05, *p < 0.01 and ***p < 0.001.

## 4. Discussion

The initiation of ESCC is associated with multiple risk factors, genes, and stages. Our previous epidemiological surveys found that N-nitrosamine disinfection by-products in drinking water contribute to 54.3% of the ESCC risk,^4^ and subsequently rat models confirmed that N-nitrosamine can initiate early carcinogenesis of ESCC.^4,22,32^ Additionally, increasing reports also have proposed that poor oral hygiene contributes to ESCC risk, although causality and independency of some indicators are uncertain.^33,34^ For instance, in inflammatory esophageal mucosa, Barrett’s esophagus has been evidenced an increase of *Prevotella* and *Actinobacillus* and a decrease of *Streptococcus;*^35^ Shao et al. revealed that cancer tissues contained more *Fusobacterium* and less *Streptococcus* than para-carcinoma tissue in ESCC patients.^36^ However, in a high concentration of N-nitrosamine exposure areas, our 16S rDNA sequencing found that *Fp*, a Gram-negative oral commensal, was significantly enriched in ESCC tissues.^3^ Environmental factors tend to favor the dominance of certain specific flora, for example, NNK combined with BaP increased *Bifidobacterium* and reduced *Acetatifactor* in initiation lung cancer.^37^ Therefore, there is a need to elucidate whether *Fp* participated and how it played synergistic effect in carcinogenic process of N-nitrosamines.

In this study, we observed that *Fp* promoted the formation of EMT-like subtypes characterized by lipid metabolism disorder through virulence gene FadAL, and in vitro exposure to N-nitrosamines significantly heightened the transcriptional activity of FadAL. However, a key limitation of our study is the inability, by design, to assess the correlation between urinary N-nitrosamine concentrations and FadAL expression in ESCC patients. N-nitrosamines are well-recognized environmental toxins that exhibit carcinogenicity, teratogenicity, and mutagenicity. It is generally accepted that the carcinogenic effects of N-nitrosamines mainly related to DNA alkylation damage,^38^ DNA methylation^39^ and abnormal expression of non-coding RNA.^40^ To date, it is largely unclear how the activation of virulence genes is mediated by N-nitrosamines. Interestingly, some studies have reported that the expression of virulence genes is affected by energy resources,^41^ such as carbon sources (glucose)^42^ and nitrogen source.^43^ Here, we found that NMBzA enhanced the transcriptional activity of FadAL, a best explanation is that NMBzA serves as a nitrogen source to regulate two component systems of *Fp*. However, this hypothesis needs to be verified by further studies.

The TME is highly heterogeneous, and some studies have deciphered the molecular subtypes of ESCC based on the somatic mutation profile and copy number variation on the genome.^44,45^ Wang et al. identified three ESCC molecular subtypes with different prognosis, characterized by glycogen metabolism downregulation, Wnt signaling pathway activation, and immunosuppression.^15^ For Wnt signaling pathway activation，however, it was activated by FadA of *Fn* in colorectal cancer.^46^ These findings suggest that microbes may be associated with the formation of specific cell clusters within the TME. Herein, our GSVA analysis also recognized 3 ESCC molecular subtypes, including EMT state, immunosuppression state and cell cycle state. It is worth noting in our study that the detection rate of *Fusobacterium* is higher in EMT state and correlated with tumor staging. Moreover, EMT state has highly active fatty acid metabolism and sphingolipid metabolism characteristics. Transcriptional reprogramming of cellular metabolism, a feature of both cancer cells and their neighboring cells in the TME, supports the bioenergetic, biosynthetic, and reduction-oxidation needs of a rapidly dividing tumor. In terms of EMT, the glutaminolysis, and accumulation of lactic acid, triglyceride, arachidonic can elicit an EMT.^47^ Additionally, Chen, Sai et al. revealed that gut microbiome and serum metabolic profile had a strong synergy, and lung cancer was induced by *Lachnospiraceae_UCG-006-*mediated L-valine reduction.^48^ Therefore, we propose that *Fp* may induce the formation of EMT subtypes by disrupting lipid metabolism processes.

Pathogens have evolved many strategies to attack the host, one of the most important ways is through virulence factor that affect host cell signaling pathways,^8^ such as W83 membrane component of *P. gingivalis* leads a strong metabolic gene changing,^49^ and cagA from *H. pylori* induces inflammatory reaction.^50^ In this study, FadAL，a virulence factor of *Fp*, is identified, and it promotes the formation of EMT subtypes by affecting the TG and PA accumulation. While FadAL contains a conserved FadA domain, its homology index with FadA of *Fn* is only 37.4%, indicating a significant functional distinction from FadA. Our subsequent results confirmed verified FadAL binding to FLOT1, initiating EMT. FLOT1, a member of the flotillin family of proteins, serves as a marker of lipid rafts that play a crucial role in tumorigenesis by regulating phagocytosis, actin cytoskeleton reorganizations and signaling transduction.^51^ It was shown that overexpression of FLOT1 induced oncogenic signaling pathways that facilitate tumor metastasis by accelerating the cell cycle and initiating EMT through PI3K/AKT, NF-Κb, and TGF-β axis.^52,53^ Moreover, FLOT1 accelerated the glucose uptake in adipocytes.^54^ In this study, we revealed that recombinant FadAL-mediated activation of FLOT1 accelerated the fatty acid accumulation, especially TG and PA. This consistency was observed when FLOT1 inhibition effectively blocked Fp infection-induced migration, invasion, and fatty acid accumulation in esophageal cancer cells. However, another limitation of our study is the unconfirmed effect of FadAL^−/−^ *Fp* infection. Due to the restrictive modification system of *Fp*, current gene knockout techniques cannot be employed for knockout experiments. FASN, the sole human lipogenic enzyme capable of de novo fatty acid synthesis, catalyzes the endogenous synthesis of fatty acids.^29^ Here, we found that, in ESCC cells, suppressing of FASN or FLOT1 almost completely attenuated intracellular TG and PA accumulation induced by recombinant FadAL or *Fp* infection. But more importantly, we also observed that in ESCC tissues, a positive correlation between FLOT1 level and FASN expression. Thus, FLOT1 is a positive regulator of lipid accumulation, and it participates in de novo fatty acid synthesis of ESCC cells by regulating FASN. To confirm this, we performed the PI3K/AKT inhibitor treatment. The results showed that inhibition of PI3K significantly suppressed the expression of FASN and SREBP1 due to FadAL treatment, suggesting that FLOT1 may also be a potential therapeutic target for *Fp* infection.

**Figure 7.**
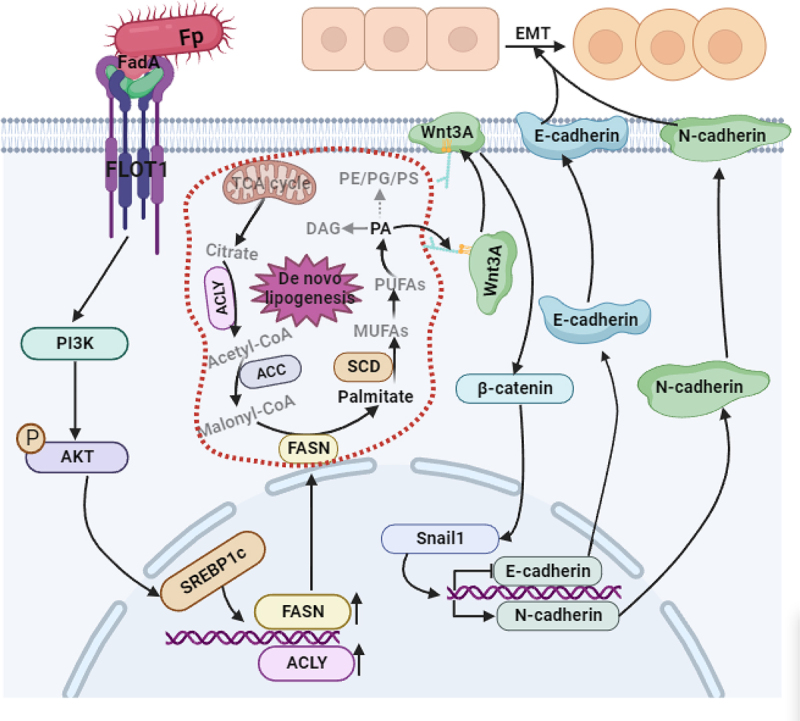
Schematic representation that FadAL binding to FLOT1 promotes the formation of EMT subtypes in *fp*-induced ESCC by modulating Wnt3a palmitoylation.

Fatty acids, as crucial components of membrane lipids, serve not only as an essential source for cellular energy metabolism but also lead to cell cytotoxicity and damage.^55^ For example, lactic acid suppressed the proliferation and cytokine production of CTLs up to 95% and led to a 50% decrease in cytotoxic activity.^56^ However, emerging evidence suggests that metabolites can regulate the epigenetic modification of proteins,^57^ for instance, S-adenosylmethionine provides methyl donor for protein methylation,^58^ and PA can modify protein palmitoylation.^24^ Palmitoylation, a reversible post-translational lipid modification, is known to regulate protein membrane localization and trafficking. As Zhang et al. reported that PA-mediated β-catenin palmitoylation and stabilization activate β-catenin axis, promoting CRC progression. In this study, we discovered that FadAL activates Wnt3A/β-catenin pathway by coordinating FLOT1/FASN axis. Differently, PA induces palmitoylation modification of Wnt3A, rather than β-catenin in ESCC, contradicting the previous study in CRC.^24^ However, these different results support the β- catenin palmitoylation exhibits high specificity in different cancer states. Some strong evidences showed that the Wnt/β-catenin activation can result in the nuclear translocation of β-catenin, triggering EMT.^59,60^ Altogether, we revealed a novel PA-induced Wnt3A modification, and the mechanism by which palmitoylation regulates Wnt3A/β-catenin activity, complementing our knowledge of canonical Wnt3A/β-catenin signaling pathway (Figure 7).

In conclusion, the current study illustrates that N-nitrosamine-mediated upregulation of FadAL induces the formation of EMT subtypes in ESCC by directly interacting with FLOT1 and promoting PA accumulation, thereby enhancing Wnt3A palmitoylation and activating the Wnt/β-catenin signaling pathway. These findings deepen our understanding of the relationship between intratumoral microbiota and tumor molecular subtypes. After further research, FadAL may become a vital chemical carcinogenic target for ESCC.

## Supplementary Material

Supplemental Material

## Data Availability

The datasets used and analyzed during the current study are available from the corresponding authors on reasonable request.
