# Supplementary material for: Synergism of Fusobacterium periodonticum and N-nitrosamines promote the formation of EMT subtypes in ESCC by modulating Wnt3a palmitoylation
Source: Gut Microbes. 2024 Aug 28;16(1):2391521. doi: 10.1080/19490976.2024.2391521 (PMC11364064; doi:10.1080/19490976.2024.2391521)
Supplement: Supplemental Material [file KGMI_A_2391521_SM9723.zip › R1-supplemental Figures.doc]

**Figure.S1**

**Figure.S1 *F. periodonticum* is enriched in EMT-subtype ESCC**

**(A)** The NMF rank survey at a rank of 2 to 6. **(B)**The rating of II-IV in the three clustering results. **(C)** The abundance of *Fusobacterium* in the three clustering results. **(D)**The rating of T2-T4 in the three clustering results. **(E)** The detection rate of Fp in the three clustering results. **(F-I)** sPLS-DA score plots differentiating samples based on metabolites comparing the F. periodonticum-infected group and blank group (F: Ec109-positive; G: Ec109-negative; H: Ec9706-positive; I: Ec9706-negative). **(J-K)** Functional annotation of differentially metabolites with Metaboanalyst 5.0, ranking by enrichment ratio (J: Ec109; K: Ec9706). **(L-M)** Heatmap a showing the differential metabolites between *F. periodonticum*-infected group and blank group (L: Ec109; M: Ec9706).

**Figure.S2**

**Figure.S2** ***Fp FadAL* induces EMT phenotype of ESCC cells**

**(A)** Conserved amino acid sequence motifs of the FadAL in *Fp*. **(B)** The Homology Index Analysis of between *Fp-FadAL and Fn-FadA.* **(C)** The Homology Index Analysis of between *F p-FadAL and Fn-FadA.*

**Figure.S3**

**Figure.S3 *Fp*-FadAL promotes Lipid accumulation of ESCC cells**

**(A)**sPLS-DA score plots differentiating samples based on metabolites comparing the 100ng/mL FadAL treatment and control group. **(B)**Volcano plot and **(C)**Heatmap showing the differential metabolites between 100ng/mL FadAL treatment and control group. **(D)** Differential metabolites (Red) in the metabolic pathways of unsaturated fatty acids. **(E)** Nile Red staining were used to verify the Lipid accumulation of ESCC cells by the fluorescence microscope.

**Figure.S4**

**Figure.S4 *FadAL*** **binds to FLOT1 to induces fatty acid accumulation by PI3K-AKT/FASN axis**

**(A-C)** Molecular docking simulations were used to assess the binding ability between FLOT1 and FadAL (A:FadAL; B:FLOT1; C:FadAL-FLOT1). **(D)** The silencing efficiency of si-FLOT1 was assessed by Western blots in Ec109 and Ec9706 cells. **(E)** The distribution of FLOT1 expression in 74 patients was described according to different expression of FLOT1 between tumor and ANT. **(F)** The silencing efficiency of si-FASN was assessed by Western blots in Ec109 and Ec9706 cells.

**Figure.S5**

**Figure.S5 *FadAL*** **binds to FLOT1 to induces fatty acid accumulation by PI3K-AKT/FASN axis**

**(A)** Transwell assays were performed to assess and quantify the migration and invasion in siFLOT1 cells with Fp infection (n=4).

**(B)** FISH staining, H&E staining and immunohistochemical staining of tumors from mice administered *Fp* treatments.
