# Supplementary material for: Synergism of Fusobacterium periodonticum and N-nitrosamines promote the formation of EMT subtypes in ESCC by modulating Wnt3a palmitoylation
Source: Gut Microbes. 2024 Aug 28;16(1):2391521. doi: 10.1080/19490976.2024.2391521 (PMC11364064; doi:10.1080/19490976.2024.2391521)
Supplement: Supplemental Material [file KGMI_A_2391521_SM9723.zip › R1-supplemental tables.doc]

Supplement Table 1 Detail information of 33 ESCC patients for detecting the relative abundance of *F. periodontium* in tissues

| Features | ESCC（n=28） |
| --- | --- |
| Age（x̅ ± S） | 67.1 ± 4.4 |
| Gender |  |
| male | 22(78.8%) |
| femal | 6(21.2%) |
| Somking |  |
| Yes | 13(46.5%) |
| No | 15(53.5%) |
| Drinking |  |
| Yes | 15 (53.5%) |
| No | 13(46.5%) |
| Stage |  |
| Ⅰ/Ⅱ | 14 (50.0%) |
| Ⅱ/IV | 12 (42.9%) |
| None | 2(7.1%) |
| Differentiation |  |
| Low differentiation | 6(21.4%) |
| Middle-High differentiation | 20 (71.4%) |
| None | 2 (7.1%) |

Supplement Table 2 Detail information of 74 patients for detecting the relative levels of FLOT1

| Features | FASN | | |  | FLOT1 | | |
| --- | --- | --- | --- | --- | --- | --- | --- |
|  | Up | Down | P |  | Up | Down | P |
| Years |  |  | 0.218 |  | 19 | 15 | 0.538 |
| <60 | 13 | 21 |  |  | 23 | 17 |  |
| ≥60 | 20 | 20 |  |  |  |  |  |
| Gender |  |  | 0.022^*^ |  |  |  | 0.179 |
| male | 29 | 26 |  |  | 29 | 26 |  |
| femal | 4 | 14 |  |  | 13 | 6 |  |
| Somking |  |  | 0.108 |  |  |  | 0.144 |
| Yes | 24 | 23 |  |  | 24 | 23 |  |
| No | 9 | 18 |  |  | 18 | 9 |  |
| Drinking |  |  | 0.419 |  |  |  | 0.025^*^ |
| Yes | 21 | 24 |  |  | 21 | 24 |  |
| No | 12 | 17 |  |  | 21 | 8 |  |
| Differentiation |  |  | 0.005^*^ |  |  |  | 0.036^*^ |
| Middle-High differentiation | 14 | 22 |  |  | 21 | 15 |  |
| Low differentiation | 13 | 3 |  |  | 14 | 2 |  |
| None | 17 | 5 |  |  | 12 | 10 |  |

Supplement Table 3 The sequences of siRNA used in this study

| Gene | Sequence |
| --- | --- |
| FLOT1 | 5′- CCUCAAUGUCAAGAGUGAA-3′ |
|  |  |
| FASN | 5′- AACCCUGAGAUCCCAGCGCUG-3′ |

Supplement Table 4 The sequences of primer used in this study

| Gene | Sequence |
| --- | --- |
| FLOT1 | Forward: CCAGGACTATTTGCACTCTTTGG |
|  | Reverse: CTTGGCTTTAGCTTCCCGGA |
| FASN | Forward: AAGGACCTGTCTAGGTTTGATGC |
|  | Reverse: TGGCTTCATAGGTGACTTCCA |
| Fp | Forward: ACTTGTATCTTTTTCATTTAT |
|  | Forward: TCAAACTCTATYTCAGGAACAA |
| 16s | Forward: ACTCCTACGGGAGGCAGCAG |
|  | Reverse: GGACTACHVGGGTWTCTAAT |
| GAPDH | Forward: TGCACCACCAACTGCTTAGC |
|  | Reverse: GGCATGGACTGTGGTCATGAG |

Supplementary Table 5. Antibodies were used in this study

| Antibodies | Source | Dilution |
| --- | --- | --- |
| FASN | Proteintech | 1:2000 |
| GADPH | Affinity Biosciences | 1:3000 |
| SREBF1 | Proteintech | 1:2000 |
| E-Cadherin | Hangzhou Huaan | 1:5000 |
| N-Cadherin | Hangzhou Huaan | 1:2000 |
| FASN | Hangzhou Huaan | 1:1000 |
| Snail | Abcam | 1:2000 |
| PI3K | Cell Signaling Technology | 1:1000 |
| AKT | Cell Signaling Technology | 1:1000 |
| P-AKT-Ser473 | Cell Signaling Technology | 1:2000 |
| SCD | Cell Signaling Technology | 1:1000 |
| ACLY | ABclonal | 1:1000 |
| ACC1 | Proteintech | 1:1000 |
| P-AKT(IHC) | AiFang Biological | 1:1000 |
| MMP9 | AiFang Biological | 1:1000 |
| Vimentin | Hangzhou Huaan | 1:500 |
| Goat anti-Rabbit | Hangzhou Huaan | 1:3500 |
| Goat anti-Mouse | Proteintech | 1:3500 |
